# Social media use disorder and loneliness: any association between the two? Results of a cross-sectional study among Lebanese adults

**DOI:** 10.1186/s40359-020-00421-5

**Published:** 2020-06-01

**Authors:** Lara Youssef, Rabih Hallit, Nelly Kheir, Sahar Obeid, Souheil Hallit

**Affiliations:** 1 Department of Nursing and Health Sciences, Notre Dame University, Notre Dame, Lebanon; 2 Faculty of Medicine and Medical Sciences, Holy Spirit University of Kaslik (USEK), Jounieh, Lebanon; 3 Faculty of Pedagogy, Holy Family University, Batroun, 5534 Lebanon; 4 Departments of Research and Psychology, Psychiatric Hospital of the Cross, Jal Eddib, Lebanon; 5 Faculty of Arts and Sciences, Holy Spirit University of Kaslik (USEK), Jounieh, Lebanon; 6 INSPECT-LB: Institut National de Santé Publique, Epidemiologie Clinique et Toxicologie, Beirut, Lebanon

**Keywords:** Social media disorder, Loneliness, Depression, Stress, Adults, Lebanon

## Abstract

**Background:**

In Lebanon, it is already established that mental disorders are prevalent among the population. Lebanese people are active users of social media platforms. To date, no study has previously explored the relationship between mental health and social media use disorder in Lebanon. The present study aims to learn more about the link between social media use disorder and loneliness among Lebanese people.

**Methods:**

This cross-sectional study was carried out between January and December 2018. It enrolled 456 residents of the community randomly selected from Lebanon’s governorates in a proportionate rate.

**Results:**

The results showed that 107 (23.7%) participants were classified as having social media use disorder. The results of a stepwise linear regression, taking the loneliness score as the dependent variable, showed that female gender compared to males (Beta = 0.42), having a secondary level of education compared to illiteracy (Beta = 0.65), higher social media use disorder (Beta = 0.03) and higher insomnia (Beta = 0.02) and alexithymia (Beta = 0.02) were significantly associated with higher loneliness.

**Conclusion:**

The present study was able to contribute to the literature and showed the association between social media use disorder and loneliness. These findings can benefit psychologists and public health practitioners in their future prevention and intervention plans.

## Background

Social media use is defined as electronically interacting with others through specific platforms, such as Instagram, Facebook and Twitter [1]. These platforms have become incredibly popular among youth and younger adults and have played a huge role in their developmental process and shaping their identity [2, 3]. Online social media platforms allow users to interact with other users through eliciting conversations, commenting on posts, uploading pictures, updating statuses and sharing geographical locations. Through these types of communication, users are able to share their thoughts, their daily activities and express their feelings [4]. However, with the current rapid growth of social media and its quick popularity, users are significantly prone to developing addictive behaviors, which in turn may have negative effects on their mental wellbeing [5].

To date, there are inconsistencies regarding the definition of “social media use disorder” [1]. “Internet use disorder” is known to be a mental health disorder with a debate on the scientific term [6]; in 2012, the American Psychiatric Association recommended that “Internet Use Disorder” should be added to the third section of the Diagnostic and Statistical Manual of Mental Disorders (DSM-5) [7]. DSM-5 identifies Internet gaming disorder as a tentative disorder [8], however, social media addiction has not been classified in the DSM-5 yet [9]. The available assessment tools are specifically based on several different diagnostic suggestions like “Internet Addiction Test” and “Internet Gaming Disorder”. Moreover, some assessment tools are focusing on one specific social media platform and disregarding others. This in turn is resulting in the lack of availability of reliable and unified information on social media use disorder [6].

Feeling lonely or “loneliness” is an emotional state that is characterized by being in an unpleasant state, where there is inconsistency in one’s desired and perceived levels of social connectedness [10]. To date, there have been two contradicting theories regarding social media use disorder and loneliness. One has advocated that time spent online replaces time spent offline (which is time spent with friends), and thus the quality of friendships is reduced [11]. This so called “displacement hypothesis” concludes that social media use increases the feeling of loneliness. On another hand, other studies have advocated that spending more time online enhances the quality of friendships, and thus the so called “stimulation hypothesis” concludes that social media use decreases loneliness [12].

Studies continued to explore social media disorder and “Problematic Internet Use” and concluded that they are associated with several psychological factors like depression, social isolation, alexithymia (defined as the inability of cognitive processing of emotions, which is accompanied by the diminished capacity to recognize and express emotions [13]) and loneliness [14–16]. The term “Problematic Internet Use” was introduced by several authors to describe internet or computer addiction or pathological use of the internet [17–22]. It is basically defined by the person’s inability to control their use of the internet [23]. However, the concept of “Problematic Internet Use” has changed over the past 20 years. During the early forms of Internet use, users merely browsed static pages to read information and/or send messages. Now, with the presence of social media platforms, the Internet became a thread that textures the fabric of our lives. It is now used to connect people from all over the world, and users spend as much time online as they do offline [7]. Davis et al. provides a cognitive behavioral model to focus on the maladaptive cognitions associated with Problematic Internet Use. In his framework, he explains how Problematic Internet Use made users more vulnerable to psychosocial problems and psychopathological disorders like depression, social anxiety, substance dependence and loneliness [16].

Based on Caplan’s model, individuals who have self-perceived social incompetence, tend to experience negative mood states like loneliness [24]. These individuals prefer communicating via online routes and engage in online social communication as an escape from their negative mood, and consequently further reinforce Internet use [24]. Studies have reported that people who excessively use the Internet, spend less time interacting face to face, which in turn results in depression and loneliness [25]. Similarly, people who experience high levels of loneliness use the Internet for emotional support [26].

Loneliness is not only associated with social media disorder, rather with other mental health problems. It has been indicated that loneliness produces stress, which in turn creates the illusion of social rejection [27]. Moreover, depression and loneliness have been intimately connected, where loneliness acted as a risk factor for developing depressive symptoms [28]. Similarly, loneliness also predicted anxiety. Studies have shown that there was a strong association between loneliness states and anxious symptoms [29].

Moving further, loneliness is also related to different sociodemographic factors. For example, studies showed that women had higher level of loneliness than men [29]. Also, being single, having lower education level and having a lower income were reported to be associated with loneliness [30].

In Lebanon, it is already established that mental disorders are prevalent among the population [31]. Also, like any other population, Lebanese people are active users of social media platforms. However, to date, no study has previously explored the relationship between mental health and social media use disorder in Lebanon. Therefore, the present study aims to learn more about the link between social media use disorder and loneliness among Lebanese adults.

## Methods

### Study design and participants

This cross-sectional study was carried out between January and December 2018. It enrolled 456 residents of the community randomly selected from Lebanon’s governorates in a proportionate rate. The governorates are divided into Caza (stratum), divided into villages. From a list provided by the Central Agency of Statistics in Lebanon, we chose two villages per Caza where the questionnaire was distributed randomly to the households, based on a random sampling technique to select the included house [19]. Those who agreed to take part in the study were invited to complete the questionnaire via a face-to-face interview. All individuals over the age of 18 were eligible to participate. Excluded were those with dementia (according to one of the family members), and those who refused to complete the questionnaire. Data collection was performed by study-independent clinical psychologists. The methodology used in this study is similar to the one used in prior publications [32].

#### Minimal sample size calculation

The G-power program was used to calculate the minimum sample size needed for our study, with an acceptable 5% margin of error, a power of 90% and an expected medium effect size (r = 0.3) of social media use on loneliness in the absence of previous Lebanese studies, the results indicated that we need 109 participants to participate in the study.

### Questionnaire

The questionnaire used during the interview was in Arabic, the native language of Lebanon. The first part assessed the sociodemographic characteristics of the participants (age, number of kids, gender, education level, socioeconomic level and marital status). The second part of the questionnaire consisted of measures used in this study as follows:

### Social media disorder scale (SMD)

The SMD is a 27-item scale that measure the degree of addiction to social media [9]. Higher scores indicated higher social media use disorder. The Cronbach’s alpha value for this scale in this study was α = 0.847.

### Toronto alexithymia scale (TAS-20)

The twenty-item TAS-20 [33] scale was used to assess alexithymia, with responses graded based on a five-point Likert scale (1 = strongly disagree to 5 = strongly agree). Higher scores indicated higher alexithymia. The Cronbach’s alpha value for this scale in this study was α = 0.862.

### Hamilton depression rating scale (HDRS)

The validated Arabic version of the HDRS was used in this study [34]. The first 17 items of the HDRS are scored and measure the severity of depressive symptoms [35]. The total depression score was calculated by summing the answers of these seventeen items. Higher scores indicated higher depression. The Cronbach’s alpha value for this scale in this study was α = 0.873.

### Hamilton anxiety scale (HAM-A)

The HAM-A [36], validated in Lebanon [37], was used scale to measure anxiety in medical and research sites. It consists of 14 items, rated according to a four-point Likert scale (0 = symptoms not present to 4 = very severe symptoms). Higher scores indicated higher anxiety. The Cronbach’s alpha value for this scale in this study was α = 0.914.

### Perceived stress scale (PSS)

It is a 10-item classic stress assessment instrument [38]. The questions in this scale ask about your feelings and thoughts during the last month, with the answers measured on a 5-point Likert scale: 0 (never) up to 4 (very often). Higher scores indicated higher perceived stress. The Cronbach’s alpha value for this scale in this study was α = 0.743.

### Lebanese insomnia scale (LIS-18)

This 18-item scale is used for the diagnosis of insomnia on the basis of several validated/universally applicable scales. Answers are graded on a 5-point Likert scale (1 = Never to 5 = Always), with higher scores indicating higher insomnia. The Cronbach’s alpha value for this scale in this study was α = 0.815.

### Jong-Gierveld loneliness scale

Subjective loneliness was assessed by the modified version of the Jong-Gierveld Loneliness Scale, composed of 5 items [39]. One point was given for a yes answer and zero was given for a no answer [39]. Higher scores would indicate more loneliness. The Cronbach’s alpha value for this scale in this study was α = 0.621.

### Statistical analysis

SPSS software version 25 was used to conduct data analysis. Cronbach’s alpha values were recorded for reliability analysis for all the scales. A descriptive analysis was done using the counts and percentages for categorical variables and mean and standard deviation for continuous measures. The Student t-test was used to compare continuous variables in two groups. Pearson correlation was used for linear correlation between continuous variables. The Student t-test was used to compare the means of 2 groups. A stepwise linear regression was conducted, taking the loneliness score as the dependent variable. All variables that showed a *p* < 0.05 in the bivariate analysis were considered as important variables to be entered in the model in order to eliminate potentially confounding factors as much as possible. A *p*-value less than 0.05 was considered significant.

## Results

The sociodemographic characteristics of the participants are summarized in Table 1. The results showed that the mean age of the participants was 27.29 ± 11.46 years and the mean number of hours spent on social media was 6.22 ± 4.92. The majority of the participants where females (61.8%), had a university level of education (66.7%), single (68.1%), with a low monthly income (61.4%). Almost all participants use their cellular as the mostly used device on social media (92.9%) and 19.4% were smokers. The mean social media use disorder scale score was 8.15 ± 5.71.

**Table 1:** sociodemographic characteristics of the sample population

	Frequency	Percentage
**Gender**		
Male	176	38.2%
Female	285	61.8%
**Education level**		
Illiterate / Primary/ Complementary	57	12.5%
Secondary	95	20.8%
University	304	66.7%
**Monthly income**		
< 1000 $	266	61.4%
1000–2000 $	122	28.2%
> 2000 $	45	10.4%
**Marital status**		
Single / Widowed / Divorced	326	70.0%
Married	140	30.0%
**Kind of device mostly used on social media**		
Laptop	22	5.2%
Cellular	394	92.9%
PC	3	0.7%
Tablet	5	1.2%
**Smoking**		
Yes	89	19.4%
No	370	80.6%
	**Mean**	**SD**
**Age (in years)**	27.29	11.46
**Number of kids**	0.76	1.55
**Number of hours spent on social media**	6.22	4.92

### Bivariate analysis

Higher mean loneliness scores were significantly found in females (vs males), in those with a secondary education level of education (vs all other education levels) and those with a low monthly income (vs all other categories). Higher alexithymia (r = 0.221), higher depression (r = 0.247), higher perceived stress (r = 0.237), higher anxiety (r = 0.163), higher social media use disorder scale score (r = 0.170) and higher insomnia (r = 0.192) were significantly associated with higher loneliness score (Tables 2 and 3).

**Table 2:** Bivariate analysis of categorical variables associated with loneliness

	Loneliness score	p-value
	Mean ± SD	
**Gender**		
Male	1.30 ± 1.45	0.005
Female	1.75 ± 1.68	
**Monthly income**		
< 1000 $	1.80 ± 1.71	0.001
1000–2000 $	1.42 ± 1.55	
> 2000 $	0.88 ± 1.21	
**Education level**		
Illiterate/ primary / complementary	1.60 ± 1.66	0.004
Secondary	2.08 ± 2.07	
University	1.41 ± 1.42	

Variables showing significant associations with the loneliness score are showing in the table; Post hoc analysis: education level (secondary vs university p = 0.002); monthly income (< 1000 USD vs > 2000 USD *p* = 0.002)

**Table 3:** Bivariate analysis of continuous variables associated with loneliness

	Loneliness	Alexithymia	Depression	Perceived stress	Anxiety	Age	Number of kids	Problematic social media use	Insomnia
**Loneliness**	1								
**Alexithymia**	0.221^a^	1							
**Depression**	0.247^a^	0.225^a^	1						
**Perceived stress**	0.237^a^	0.280^a^	0.282^a^	1					
**Anxiety**	0.163^b^	0.271^a^	0.499^a^	0.343^a^	1				
**Age**	−0.021	−0.199^a^	−0.002	−0.011	− 0.08	1			
**Number of kids**	−0.054	−0.114^c^	0.04	0.007	0.002	0.706^a^	1		
**Problematic social media use**	0.170^a^	0.349^a^	0.129^b^	0.092	0.178^a^	−0.307^a^	−0.181^a^	1	
**Insomnia (LIS scale)**	0.192^a^	0.139^b^	0.442^a^	0.270^a^	0.491^a^	0.048	0.028	0.091	1

^a^*p* < 0.001; ^b^*p* < 0.01; ^c^*p* < 0.05

### Multivariable analysis

The results of a stepwise linear regression, taking the loneliness score as the dependent variable, showed that female gender compared to males (Beta = 0.42), having a secondary level of education compared to illiteracy (Beta = 0.65), higher social media use disorder (Beta = 0.03) and higher insomnia (Beta = 0.02) and alexithymia (Beta = 0.02) were significantly associated with higher loneliness (Table 4). DISCUSSION.

**Table 4:** Multivariable analysis: Linear regression taking the loneliness score as the dependent variable

	Unstandardized Beta	Standardized Beta	p-value	95% Confidence interval	
				Lower bound	Upper bound
Alexithymia	0.02	0.13	0.012	0.01	0.04
Insomnia	0.02	0.17	< 0.001	0.01	0.03
Social media use disorder score	0.03	0.11	0.028	0.003	0.06
Secondary education level compared to illiteracy*	0.65	0.16	0.001	0.27	1.02
Gender (females vs males*)	0.42	0.13	0.007	0.12	0.73

Variables entered: Social media use disorder score, gender, anxiety (HAMA scale), alexithymia (TAS-20 scale), insomnia (LIS scale), monthly income and education level

*Reference group

Whether or not social media use disorder is a direct cause of loneliness remains controversial, however, our study was able to show a strong association between the two, in addition to other mental health illnesses. Our results have shown that in addition to some sociodemographic factors (being a female and having secondary level education), higher insomnia, alexithymia and social media use disorder were associated with higher levels of loneliness.

Although our study did not measure the same components included in Caplan’s cognitive behavioral model, our findings support the model’s theory. This is specifically relating to negative mood states, such as stress and depression, leading people to the irregular use of online platforms in the aim of mood regulation [24].

Moreover, the association between insomnia and loneliness obtained in this study can be explained by the fact that people who spend long hours awake, experience the feeling of isolation. This in turn demotivates them to engage with others and build social connections, and thus the feeling of loneliness is amplified [40]. On the other hand, several studies have reported that loneliness itself leads to sleep fragmentation and poor sleep quality [41, 42].

Furthermore, similar to results reported in the literature, alexithymia was found to be correlated with loneliness [43]. People with alexithymia have difficulty in expressing their emotions and thus they have fewer close relationships, poor social support networks and often feel disconnected from others. All these factors amplify the feeling of loneliness [44]. To the best of our knowledge, only one study in the literature measured the direct effect of alexithymia on loneliness and found out that this strong association was a result of lack of trust in others [45].

While our findings come in line with many results from other studies, the present evidence in the literature is quite wide and contradicting. Based on Robert Weiss’ theory of loneliness, individuals who spend more time online have higher chances of emotional loneliness but lower chances of social loneliness [46]. Our results were able to show the emotional loneliness side, where individuals use social media as the primary route of communication, and thus it sabotages face to face social activities and eliminates “strong ties” [47]. We were also able to challenge the assumption that social media communication can effectively provide people with their social needs satisfied by face to face communication [7]. There is a vivid relationship between social media use and loneliness, where higher usage of social media showed higher level of loneliness among users [48]. Our results indicated this to be true even after adjusting for other interfering factors associated with loneliness (i.e. stress, anxiety and depression).

Although we were able to highlight the effect between social media use disorder and loneliness, some limitations need to be highlighted. There is a need to further explore the type of applications used, the time spent online and the reasons these social media platforms are being used for [49]. In some studies, using social media platforms to communicate with relatives was associated with lower levels of social loneliness, therefore, the nature of this use needs to be closely examined [50]. Other factors to be taken into consideration may be the place of residence (at home or in a dorm), which could have an influence on the social media use and addiction [51]. Similarly, whether you are using it to gamble, meet new people or simply do research has a huge effect. In other studies, people who were using the internet to meet other people had higher levels of loneliness than those who used it for research and homework [52, 53]. Also, the age of starting to use social media platforms, and the ease of access should not be neglected when studying association with loneliness [51, 54]. Despite showing good reliability values in this study, the scales used in the study, except the HDRS, HAM-A and LIS-18, are not validated in Lebanon. Finally, one should not omit to mention the strengths of this study. Knowing the complex psychological conditions of studying depression and loneliness, we did not overlook the influence of other factors such as age, gender and education [7]. Finally, it is important to stress that future research questions should focus on investigating different types of social media platforms and their effect on users’ habits maintenance and how moderators (like number of friends on Facebook, number of followers on Instagram, number of picture likes, etc.) are associated with this addiction [55].

## Conclusion

The present study was able to contribute to the literature and showed the association between social media use disorder and loneliness. These findings can benefit psychologists and public health practitioners in their future prevention and intervention plans. People who negatively use social media platforms can then be offered with appropriate counselling and coping mechanisms. Future research should further focus on the specific uses of social media, predispositions and its effect on loneliness. Future longitudinal studies should also try to reveal the temporality issue to show whether young people start using social media because they feel lonely and depressed or whether they become more lonely and depressed with increasing use of social media.

## Data Availability

The authors do not have the right to share any data information as per their institutions policies.

## Abbreviations

DSM-5: Diagnostic and Statistical Manual of Mental Disorders, Fifth edition
SMD: Social media disorder
TAS: Toronto Alexithymia Scale
HDRS: Hamilton depression rating scale
HAM-A: Hamilton anxiety scale
PSS: Perceived Stress Scale
LIS: Lebanese Insomnia Scale

## References

[1] Michikyan, M. and C. Suárez-Orozco, Adolescent media and social media use: Implications for development. 2016, SAGE publications Sage CA: Los Angeles, CA.

[2] Bartsch, M. and K. Subrahmanyam, *Technology and self-presentation.* The Wiley Handbook of Psychology, Technology, and Society. Hoboken, NJ: Wiley-Blackwell, 2015: p. 339–357.

[3] Subrahmanyam, K. and D. Smahel, *Digital youth: The role of media in development*. 2010: Springer Science & Business Media.

[4] Kaplan AM, Haenlein M (2010). Users of the world, unite! The challenges and opportunities of social media. Business horizons.

[5] Shettar M (2017). Facebook addiction and loneliness in the post-graduate students of a university in southern India. Int J Soc Psychiatry.

[6] Bányai, F., et al., *Problematic social media use: Results from a large-scale nationally representative adolescent sample.* PLoS One, 2017. **12**(1). 10.1371/journal.pone.0169839 PMC5222338 28068404

[7] Yao MZ, Zhong Z-J (2014). Loneliness, social contacts and internet addiction: a cross-lagged panel study. Comput Hum Behav.

[8] Association, A.P., *Diagnostic and statistical manual of mental disorders (DSM-5®)*. 2013: American Psychiatric Pub. 10.1590/s2317-17822013000200017 24413388

[9] Van Den Eijnden RJ, Lemmens JS, Valkenburg PM (2016). The social media disorder scale. Comput Hum Behav.

[10] Heinrich LM, Gullone E (2006). The clinical significance of loneliness: a literature review. Clin Psychol Rev.

[11] Kraut R (1998). Internet paradox: a social technology that reduces social involvement and psychological well-being?. Am Psychol.

[12] Shaw LH, Gant LM (2002). Users divided? Exploring the gender gap in internet use. CyberPsychology & Behavior.

[13] Taylor GJ, Bagby RM (2004). New trends in alexithymia research. Psychother Psychosom.

[14] Ceyhan AA, Ceyhan E (2008). Loneliness, depression, and computer self-efficacy as predictors of problematic internet use. CyberPsychology & Behavior.

[15] Chou C, Hsiao M-C (2000). Internet addiction, usage, gratification, and pleasure experience: the Taiwan college students’ case. Comput Educ.

[16] Davis RA (2001). A cognitive-behavioral model of pathological internet use. Comput Hum Behav.

[17] Bai Y-M, Lin C-C, Chen J-Y (2001). Internet addiction disorder among clients of a virtual clinic. Psychiatr Serv.

[18] Beard KW, Wolf EM (2001). Modification in the proposed diagnostic criteria for internet addiction. Cyberpsychology & behavior.

[19] Belsare TJ, Gaffney GR, Black DW (1997). Compulsive computer use. Am J Psychiatr.

[20] Griffiths M (1991). The observational study of adolescent gambling in UK amusement arcades. J Community Appl Soc Psychol.

[21] OReilly M (1996). Internet addiction: a new disorder enters the medical lexicon. CMAJ: Canadian Medical Association journal.

[22] Young KS (1998). Internet addiction: the emergence of a new clinical disorder. Cyberpsychology & behavior.

[23] Shapira NA (2000). Psychiatric features of individuals with problematic internet use. J Affect Disord.

[24] Caplan SE (2003). Preference for online social interaction: a theory of problematic internet use and psychosocial well-being. Commun Res.

[25] Ybarra ML, Alexander C, Mitchell KJ (2005). Depressive symptomatology, youth internet use, and online interactions: a national survey. J Adolesc Health.

[26] Morahan-Martin J, Schumacher P (2003). Loneliness and social uses of the internet. Comput Hum Behav.

[27] Cacioppo JT, Hawkley LC, Berntson GG (2003). The anatomy of loneliness. Curr Dir Psychol Sci.

[28] Yaacob, S.N., et al., *Loneliness, stress, self esteem and depression among Malaysian adolescents.* Jurnal Kemanusiaan, 2009. **7**(2).

[29] Chang EC (2018). Relationship between loneliness and symptoms of anxiety and depression in African American men and women: evidence for gender as a moderator. Personal Individ Differ.

[30] Theeke LA (2010). Sociodemographic and health-related risks for loneliness and outcome differences by loneliness status in a sample of US older adults. Res Gerontol Nurs.

[31] Karam G (2016). Prevalence, correlates, and treatment of mental disorders among Lebanese older adults: a national study. Am J Geriatr Psychiatry.

[32] Youssef L, et al. Social media use disorder and alexithymia: Any association between the two? Results of a cross‐sectional study among Lebanese adults. Social media use disorder and alexithymia: Any association between the two? Results of a cross‐sectional study among Lebanese adults. Perspect Psych Care 2020. 10.1111/ppc.12506. 10.1111/ppc.12506 32239534

[33] Bagby RM, Parker JD, Taylor GJ (1994). The twenty-item Toronto alexithymia scale--I. item selection and cross-validation of the factor structure. J Psychosom Res.

[34] Obeid S (2018). Validation of the Hamilton depression rating scale (HDRS) and sociodemographic factors associated with Lebanese depressed patients. Encephale.

[35] Hamilton M (1960). A rating scale for depression. J Neurol Neurosurg Psychiatry.

[36] Hamilton M (1959). The assessment of anxiety states by rating. Br J Med Psychol.

[37] Hallit S (2019). Validation of the Hamilton anxiety rating scale and state trait anxiety inventory a and B in Arabic among the Lebanese population. Clinical Epidemiology and Global Health.

[38] Cohen S, Kamarck T, Mermelstein R (1983). A global measure of perceived stress. J Health Soc Behav.

[39] Wilson RS (2007). Loneliness and risk of Alzheimer disease. Arch Gen Psychiatry.

[40] Hom MA (2017). Investigating insomnia as a cross-sectional and longitudinal predictor of loneliness: findings from six samples. Psychiatry Res.

[41] Harris RA, Qualter P, Robinson SJ (2013). Loneliness trajectories from middle childhood to pre-adolescence: impact on perceived health and sleep disturbance. J Adolesc.

[42] Kurina LM (2011). Loneliness is associated with sleep fragmentation in a communal society. Sleep.

[43] NOORI SS, Nargesi F (2013). The relationship of alexithymia with loneliness and comparison of them in male and female students.

[44] Frye-Cox NE, Hesse CR (2013). Alexithymia and marital quality: the mediating roles of loneliness and intimate communication. J Fam Psychol.

[45] Qualter P (2009). Loneliness, interpersonal distrust, and alexithymia in university students 1. J Appl Soc Psychol.

[46] Weiss RS (1973). Loneliness: the experience of emotional and social isolation.

[47] Moody EJ (2001). Internet use and its relationship to loneliness. CyberPsychology & Behavior.

[48] Wang K (2018). Active public Facebook use and adolescents' feelings of loneliness: evidence for a curvilinear relationship. J Adolesc.

[49] Gross EF, Juvonen J, Gable SL (2002). Internet use and well-being in adolescence. J Soc Issues.

[50] Sum S (2008). Internet use and loneliness in older adults. CyberPsychology & Behavior.

[51] Kormas G (2011). Risk factors and psychosocial characteristics of potential problematic and problematic internet use among adolescents: a cross-sectional study. BMC Public Health.

[52] Cao H (2011). Problematic internet use in Chinese adolescents and its relation to psychosomatic symptoms and life satisfaction. BMC Public Health.

[53] Jang KS, Hwang SY, Choi JY (2008). Internet addiction and psychiatric symptoms among Korean adolescents. J Sch Health.

[54] Hur MH (2006). Demographic, habitual, and socioeconomic determinants of internet addiction disorder: an empirical study of Korean teenagers. Cyberpsychology & behavior.

[55] Hunt MG (2018). No more FOMO: limiting social media decreases loneliness and depression. J Soc Clin Psychol.

