# Correction to: Social media use disorder and loneliness: any association between the two? Results of a cross-sectional study among Lebanese adults

**DOI:** 10.1186/s12888-020-02740-8

**Published:** 2020-06-23

**Authors:** Lara Youssef, Rabih Hallit, Nelly Kheir, Sahar Obeid, Souheil Hallit

**Affiliations:** 1 Department of Nursing and Health Sciences, Notre Dame University, Notre Dame, Lebanon; 2 Faculty of Medicine and Medical Sciences, Holy Spirit University of Kaslik (USEK), Jounieh, Lebanon; 3 Faculty of Pedagogy, Holy Family University, Batroun, 5534 Lebanon; 4 Departments of Research and Psychology, Psychiatric Hospital of the Cross, Jal Eddib, Lebanon; 5 Faculty of Arts and Sciences, Holy Spirit University of Kaslik (USEK), Jounieh, Lebanon; 6 INSPECT-LB: Institut National de Santé Publique, Epidemiologie Clinique et Toxicologie, Beirut, Lebanon

**Correction to: BMC Psychology (2020) 8:56**

**https://doi.org/10.1186/s40359-020-00421-5**

Following publication of the original article [1], the authors identified a missing reference.

Hallit, S., Sacre, H., Haddad, C. et al. Development of the Lebanese insomnia scale (LIS-18): a new scale to assess insomnia in adult patients. BMC Psychiatry 19, 421 (2019). 10.1186/s12888-019-2406-y
